# Food-Anticipatory Behavior in Neonatal Rabbits and Rodents: An Update on the Role of Clock Genes

**DOI:** 10.3389/fendo.2018.00266

**Published:** 2018-05-24

**Authors:** Mario Caba, Jorge Mendoza

**Affiliations:** ^1^Centro de Investigaciones Biomédicas, Universidad Veracruzana, Xalapa, Mexico; ^2^Institute of Cellular and Integrative Neurosciences, CNRS UPR-3212, University of Strasbourg, Strasbourg, France

**Keywords:** circadian rhythms, clock gene mutant, restricted feeding, food entrainment, corticosterone, PER1 protein

## Abstract

In mammals, the suprachiasmatic nucleus (SCN), the master circadian clock, is mainly synchronized to the environmental light/dark cycle. SCN oscillations are maintained by a molecular clockwork in which certain genes, Period 1–2, Cry1–2, Bmal1, and Clock, are rhythmically expressed. Disruption of these genes leads to a malfunctioning clockwork and behavioral and physiological rhythms are altered. In addition to synchronization of circadian rhythms by light, when subjects are exposed to food for a few hours daily, behavioral and physiological rhythms are entrained to anticipate mealtime, even in the absence of the SCN. The presence of anticipatory rhythms synchronized by food suggests the existence of an SCN-independent circadian pacemaker that might be dependent on clock genes. Interestingly, rabbit pups, unable to perceive light, suckle milk once a day, which entrains behavioral rhythms to anticipate nursing time. Mutations of clock genes, singly or in combination, affect diverse rhythms in brain activity and physiological processes, but anticipatory behavior and physiology to feeding time remains attenuated or unaffected. It had been suggested that compensatory upregulation of paralogs or subtypes genes, or even non-transcriptional mechanisms, are able to maintain circadian oscillations entrained to mealtime. In the present mini-review, we evaluate the current state of the role played by clock genes in meal anticipation and provide evidence for rabbit pups as a natural model of food-anticipatory circadian behavior.

## Introduction

The suprachiasmatic nucleus (SCN), located in the ventral forebrain lateral to the third ventricle, is the master circadian pacemaker necessary for the control of endogenous physiological and behavioral rhythms in mammals (1). At the cellular level, a group of genes, known as clock genes, are necessary to generate and sustain circadian rhythms controlled by the SCN. This clock mechanism is a transcription–translation autoregulatory feedback loop with the positive arm comprised of *Clock* and *Bmal1* genes and their proteins. CLOCK and BMAL1 proteins form heterodimers that bind to E-box enhancer elements in the promoter region of the Period (*Per1–2*) and Cryptochrome (*Cry1–2*) genes to activate their transcription. In turn, PER and CRY proteins constitute the negative arm of the loop. CLOCK and *Bmal1* also activate the transcription of retinoic orphan receptor α, β, and REV-ERBα,β,γ, which form an auxiliary loop driving rhythmic Bmal1 transcription with activating and repressing actions, respectively.

In mammals, light is the main entraining signal for circadian rhythms. However, food also can be a synchronizer. When rats are fed one meal per day, within a few days they display “food-anticipatory activity” [FAA (2–4)], with arousal and an increase in locomotor behavior occurring some hours before mealtime. August Forel was the first to describe this phenomenon around one century ago, showing that bees anticipate the time of morning meals (4). In rats, in addition to increase in locomotor behavior, there is also an increase in serum levels of corticosterone and core body temperature before mealtime (5).

Food-anticipatory activity exhibits circadian properties such as limits of entrainment close to 24 h, transient cycles following phase shifts and persistence during fasting conditions [Rev (3)]. Following the discovery of the SCN as the locus of the master circadian clock, whether the SCN also served as the neural substrate of FAA was explored. Surprisingly, the anticipatory increase in locomotor activity, core body temperature, and corticosterone in food-entrained rats was not abolished by lesions of the SCN (5). This and subsequent experiments led to a search for the existence of a food-entrainable oscillator (3) distinct from the SCN light-entrainable oscillator. Many neural loci and glands were examined as potential sites regulating FAA, including the adrenal gland, several brain regions in the hypothalamus (i.e., ventromedial, dorsomedial and paraventricular nuclei, lateral preoptic area), the parabrachial nuclei, olfactory bulb (OB), hippocampal formation, cerebellum, amygdala and nucleus accumbens, among others [Rev (6, 7)]. In all cases, lesions or excision failed to abolish FAA. These studies suggested that FAA lies in a specific, unknown locus or, perhaps more likely, consists of an organized, distributed system of interacting structures both at the central and peripheral levels (7). In the present overview, we provide evidence for a role of clock genes in FAA, providing a new strategy to explore this phenomenon.

## Circadian Genes Relevant to Food Anticipation

Clock genes serve as key elements for the generation of circadian oscillations in the SCN. When one of these elements is rendered non-functional, alterations in behavioral and physiological rhythms appear. Because FAA is under the control of a circadian mechanism, it is logical to hypothesize that clock gene mutations might also affect FAA (Table 1).

**Table 1:** Summary of the effects of clock genes mutations in circadian locomotor activity rhythms and food-anticipatory activity (FAA).

Clock gene	Circadian alteration (locomotion)	FAA (locomotion, wheel-running)	Reference
*ClockΔ19*	Arrhythmic in DD	Normal	Pitts et al. (8)
*Npas2*	Normal	Delayed	Dudley et al. (9)
*Bmal1*	Arrhythmic in DD	Normal	Bunger et al. (10) and Pendergast et al. (11)
*Bmal1* (brain-specific)	Shortened period in DD	Attenuated	Mieda and Sakurai (12)
*Per1*	Shortened period in DD	Normal	Zheng et al. (13) and Feillet et al. (14)
*Per2^Brdm1^*	Arrhythmic in DD	Absent/normal	Zheng et al. (15), Feillet et al. (14), and Pendergast et al. (16)
*Per2* (liver-specific)	Normal in DD	Absent	Chavan et al. (17)
*Cry1–2*	Arrhythmic in DD	Attenuated	van der Horst et al. (18) and Iijima et al. (19)
*Rev-erb*α	Shortened period in DD	Attenuated	Preitner et al. (20) and Delezie et al. (21)

One of the first studies pointing to a role for clock genes in FAA comes from studies of *ClockΔ19* gene mutant mice (8). The circadian locomotor behavior of these mice is arrhythmic when animals are exposed to constant darkness (DD) conditions (22, 23). When exposed to restricted-feeding schedules, *ClockΔ19* mice show strong FAA and its persistence during food deprivation indicates that the *Clock* gene is not necessary for FAA. While this study suggested that *Clock* is not essential for FAA, it was later shown that the *Npas2* gene can compensate for the loss of *Clock*, acting as a positive transcription factor in the SCN to maintain circadian oscillations (24). This finding suggested that *Npas2* might be implicated in the regulation of FAA. Indeed, *Npas2* mutant mice exposed to temporally restricted feeding show altered FAA, requiring two or three more days to develop FAA relative to WT animals (9). Thus, *Npas2* appears to be an important gene in the regulation of FAA. However, FAA does not disappear entirely in *Npas2* mutant mice, indicating that other genes contribute to the maintenance of FAA.

As mentioned previously, the positive loop of the clockwork also includes *Bmal1*, a gene that is rhythmically expressed in the SCN and other peripheral oscillators (25). Global mutations of *Bmal1* lead to arrhythmic behavior when animals are in DD conditions (10), while FAA is normal in these animals (11). However, in one study, *Bmal1* deletions confined to the dorsomedial hypothalamus eliminated FAA (26). The reason for this discrepancy is not readily clear; because methods and protocols to measure locomotor activity differ between studies, this conclusion remains to be confirmed (26, 27). Importantly, in another study using mice with a nervous system-specific deletion of *Bmal1*, excluding the SCN clock, it was demonstrated that FAA is strongly affected, suggesting the necessity of *Bmal1* in an extra-SCN brain locus for FAA (12). Further confirming a role for *Bmal1* in FAA, in *Rev-erb*α-mutant mice exposed to restricted-feeding schedules, FAA was negatively affected (21). *Rev-erb*α is a transcription factor with a repressor activity on *Bmal1* (20).

With regard to the negative arm of the clockwork, double *Cry* gene mutations (*Cry1–2*) lead to arrhythmic behavior in mice held under DD (18) and FAA is markedly reduced (19). *Per* genes (1–2), also important components of the negative loop, are essential in the control of circadian rhythmicity. These genes are expressed rhythmically in diverse brain structures and peripheral organs [liver, heart, and lung (13, 15, 28)]. Whereas *Per1^−/−^* mutants show normal FAA, FAA is absent in *Per2^Brdm1^*-mutant mice (14). However, in a more recent examination of the same *Per2* mutant mice, FAA was not altered (16). Thus, the effects of global mutations of *Per2* on FAA remain to be clarified. Interestingly, when *Per2* is knock down specifically in the liver, FAA is totally eliminated and can be rescued by viral overexpression of liver *Per2* (17). This study indicates that FAA is not only dependent upon the brain but that it also requires normal *Per2* expression in the liver for its manifestation, confirming that *Per2* is likely an important component of the molecular mechanisms of FAA (Table 1).

Most studies of FAA examine rodents under a schedule of food restriction. However, most animals in their natural environments do no experience food restriction on a circadian schedule. In contrast, rabbit pups are fed for brief periods on a circadian schedule in nature and the lab. Thus, in the present contribution, we present evidence that supports notion that the rabbit pup constitutes a natural model of food entrainment.

## FAA in the Rabbit

Rabbit pups are born altricial, they have no fur, their eyelids and outer ears are sealed, and they remain in the maternal burrow in darkness for the first 2 weeks of their life (29). Behavioral studies (29, 30) confirm that shortly after parturition the mother leaves the nest and returns every day with a circadian periodicity to nurse pups whether they are maintained in continuous light or in light–dark conditions (31, 32). Although parturition occurs throughout the day, the time of nursing is rapidly established on lactation day 1 and then nursing occurs every 24 h at around the same hour every night, 03:52 h across lactation days 1–15 (33, 34).

## Locomotor Behavior

Although pups are not entrained by the light–dark cycle (their eyes do not open until postnatal day 10) (35), they receive periodic time cues through feeding. Every day at around the same time they ingest up to 35% of their body weight in milk (36) in around 5 min (31, 32). Hence food, in this case milk, seems to be a potent zeitgeber for rabbit pups. To explore in detail behavioral, physiological, and neural consequences of timed feeding, we scheduled nursing at two different hours, at 10:00 a.m. and at 02:00 a.m. (i.e., during the day and during the night, respectively) from postnatal (PD) 1. At PD3 (02:00 a.m. group) and PD4 (10:00 a.m. group), despite their altricial condition, pups show a significant increase in locomotor behavior 2 h before the mother’s arrival. Immediately after suckling, locomotor behavior decreases and pups remain inactive and huddled in the nest. Moreover, this locomotor increase persists for 2 days in nurse-deprived pups at the same hour of the last nursing (37).

## Corticosterone

**Figure 1:**
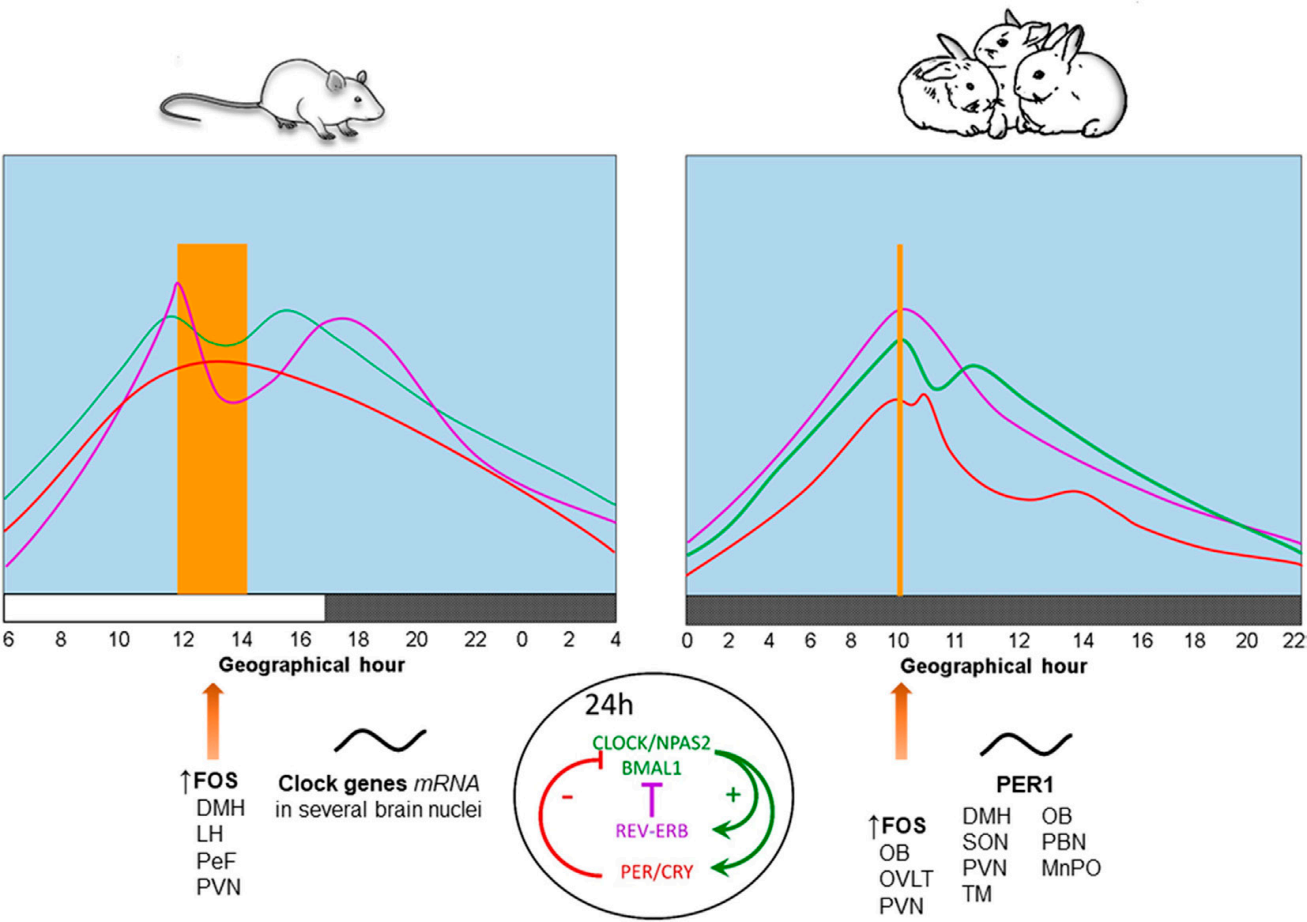
Physiological, behavioral, and neural changes associated with food-anticipatory activity and the molecular clockwork. Daily rhythms of locomotor activity (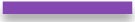), corticosterone (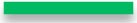), and body temperature (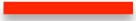) increase in anticipation of the time of feeding in both species. The molecular clock is comprised of two principal feedback loops for the expression of clock genes. In the positive loop (green) the proteins CLOCK/NPAS2 and BMAL1 act on the transcription sites of *Per, Cry*, and *Rev-erb*α genes to induce their mRNA expression. Once the final proteins of PER and CRY (negative loop; red) are produced, these have the ability to repress their own transcription *via* an inhibitory action on the *Clock-Npas2/Bmal1* dimer. The REV-ERBα protein is a transcriptional repressor for the *Bmal1* gene driving rhythmic *Bmal1*. FOS protein expression and rhythms of clock genes and proteins in several brain nuclei synchronize to mealtime. DMH, dorsomedial hypothalamic nucleus; LH, lateral hypothalamus; MnPO, median preoptic nucleus; OB, olfactory bulb; OVLT, organum vasculosum of lamina terminalis; PeF, perifornical nucleus; paraventricular nucleus; SON, supraoptic nucleus; TM, tuberomammillary nucleus. Vertical bar and big arrow, feeding time. Figure derived from data previously published by: Angeles-Castellanos et al. (43), Caba et al. (37), Escobar et al. (44), Honma et al. (42), Jilge et al. (45), Mistlberger (7), Morgado et al. (40, 41, 46), and Rovirosa et al. (39).

In contrast to neonatal rodents which are in a stress hyporesponsive period (38), we found that 7- to 9-day-old rabbits exhibit rhythmic secretion of corticosterone with higher plasma levels at the time of nursing, reaching a nadir 12 h later and increasing again in advance of the next nursing bout (39). Peak levels of corticosterone shift in parallel with the nursing schedule either during the day or the night and persist during fasting conditions (40, 41), indicating entrainment by time of nursing. In adult rodents this hormone reaches a peak at the time of food presentation (5, 42). See Figure 1.

## Core Body Temperature

Rabbit pups maintained in constant dim light exhibit a 24-h rhythm of core body temperature with a significant anticipatory rise of 0.4–0.6°C around 3 h before daily nursing. This increase is followed by a secondary postprandial rise, followed within 1–3 h by a temperature drop. Moreover, during a 48-h fast, the anticipatory rise in temperature persists, while the postprandial increase in temperature does not (45, 47). These results indicate that the anticipatory increase is endogenous and entrained by the timing of nursing, whereas the postprandial increase is induced by food ingestion. In Figure 1, we present a comparison of daily rhythms of locomotor activity, corticosterone release and body temperature in relation to FAA in adult rodents and rabbit pups. In these species, there are changes in FOS protein, clock genes, and PER1 protein in some brain structures described further below.

## Clock Genes and FAA in the Rabbit Pup

### Olfactory Bulb

Postnatal day 7 pups receive temporal time cues through the brief daily visit by their mother and ingestion of a meal once a day. To successfully ingest milk, pups depend on their OB to detect the emission of a mammary pheromone (48) and to grasp the nipple; anosmic pups are unable to suckle milk and will die of starvation (49). At PD7, rhythms of the clock genes *Per1, Bmal1*, and *Cry1* are already established in the OB, whereas a clear rhythm is not detected until PD45 in the SCN (50). The earlier maturation of the clockwork in the OB is consistent with the dependence on suckling at this age. Analysis of PER1 protein in the OB has been explored to determine the pattern of rhythms in this protein relative to the timing of suckling. At PD7, neonatal rabbits express robust rhythms of PER1 in layers of both main and accessory OBs that shift in parallel to the timing of suckling (i.e., either during the day or during the night). Moreover, PER1 expression persists during fasting conditions. Additionally, significant increases in FOS protein were detected at the time of suckling (i.e., during FAA), suggesting that the OB has a clock mechanism that anticipates nursing (51). This finding is consistent with previous work showing that the OB has an SCN-independent circadian pacemaker (52).

A milk/nipple stimulus appears to be important for OB oscillations. In this regard, the role of a mammary pheromone has been explored as an entraining signal (50); however, its importance remains unclear as the pheromone was applied at concentrations far beyond the effective concentration to elicit the oral nipple grasping response (53). Additionally, food has been explored as the entraining signal. In neonatal rabbits, the intragastric infusion of milk formula at PD7 once during the day or during the night without any maternal contact entrained rhythms of locomotor behavior and CORT, with peak values at the time of FAA. The milk stimulus also entrained rhythms of PER1 in hypothalamic nuclei (see below). These rhythms shift in parallel to the timing of milk formula infusion, demonstrating that food, in this case milk, is sufficient to entrain behavioral, physiological, and neural parameters in the neonatal rabbit (46), similar to what is seen in nursed pups. In contrast, the mammary pheromone is likely necessary for nipple detection, but not FAA.

### Suprachiasmatic Nucleus

In the SCN, there is a rhythm of PER1 in nursed and fasted pups fed either during the day or the night from PD1 (37). More importantly, there is a shift in PER1 peak expression of 2.5 h between day and night nursed pups, suggesting an entraining effect of timed nursing on the pup’s SCN. A larger shift of *Per1, Per2*, and *Bmal1* rhythms was demonstrated by shifting the time of nursing from PD4–PD7 (54). However, in this same study, there was a spontaneous advance in *Per1* of around 7 h from PD3 to PD9 in pups nursed at the same time since birth. Therefore, it is not clear if the influence of ontogenetic development of the SCN on the shift in clock genes is mediated by the timing of nursing. Although retinal projections are present in the SCN at birth, the nucleus is immature in its response to a light pulse until PD12 (55). Despite methodological differences, it is possible that the pups’ SCN is sensitive to non-photic cues. The effect of food restriction on the SCN has been reported in adult rats and may be involved in the neural mechanism of food entraining (56), although, as already mentioned, this nucleus is not essential for FAA.

### Other Brain Structures

In the dorsomedial hypothalamic nucleus (DMH) there is a complete phase shift of PER1 in parallel to a change in the time of nursing that persists in fasted pups (37). These results agree with publications in rodents (57, 58), indicating that the DMH might play an important role in food entrainment, although not as the unique brain structure regulating FAA (17). PER1 has been also analyzed in the median preoptic nucleus (MnPO), organum vasculosum of lamina terminalis, and medial preoptic area (59). However, a robust rhythm of PER1 is only detected in the MnPO at the time of FAA, a rhythm that persists during fasting. To our knowledge, there are no reports regarding a role of the MnPO in FAA in rodents, pointing to a need for further exploration. In the brainstem the dorsal vagal complex (DVC) and parabrachial nucleus (PBN) express PER1 in neonatal rabbits. Whereas the DVC shows rhythms related to food ingestion, the PER1 rhythm was entrained by milk intake in the PBN, a rhythm that persists during fasting (60). It is possible that changes in PER1 are due to food ingestion as the paraventricular, supraoptic, and tuberomammillar nuclei shows PER1 rhythms that shift in parallel to the timing of intragastric milk formula infusion (46).

## Metabolic and Hormonal Signals and the Reward System

Metabolic fuels such as glycogen and free fatty acids follow a rhythm associated with the full and empty stomach to maintain stable glucose levels; those levels are maintained even in fasting conditions (40, 41). Interestingly, the orexigenic hormone, ghrelin, which acts on the arcuate nucleus, also follows a rhythm with peak levels 12 h after the last nursing, likely participating in triggering the next FAA episode (40, 46). Indeed, in rats under restricted feeding, plasma ghrelin levels peak before mealtime (61) and, in combination with leptin, modulates the reward circuitry by acting on dopaminergic neurons in the ventral tegmental area to reinforce FAA (62, 63).

## Conclusion

Food-anticipatory activity is the expression of a circadian phenomenon in different species, usually studied in adult subjects. Here, we demonstrate that the neonatal rabbit circadian system is an ideal natural model to study the brain and molecular mechanism of FAA. FAA depends, in part, on some clock genes expressed in a circadian network of brain structures, oscillating in synchrony, and coordinated by the SCN. Combining information on brain clock gene expression in rabbit pups with mouse models of clock gene mutations for the study of FAA will help increase understanding of the molecular mechanisms implicated in food anticipation in the wild.

## Author Contributions

MC and JM contributed to the writing of the manuscript and approved the final version.

## Conflict of Interest Statement

The authors declare that the research was conducted in the absence of any commercial or financial relationships that could be construed as a potential conflict of interest. The reviewer OV and handling Editor declared their shared affiliation.
